# Formation of upd(7)mat by trisomic rescue: SNP array typing provides new insights in chromosomal nondisjunction

**DOI:** 10.1186/s13039-017-0329-1

**Published:** 2017-07-25

**Authors:** Sandra Chantot-Bastaraud, Svea Stratmann, Frédéric Brioude, Matthias Begemann, Miriam Elbracht, Luitgard Graul-Neumann, Madeleine Harbison, Irène Netchine, Thomas Eggermann

**Affiliations:** 1 INSERM, UMR_S 938, CDR Saint-Antoine, F-75012 Paris, France; 2 UMR_S 938, CDR Saint-Antoine, Sorbonne Universities, UPMC Univ Paris, 06 Paris, France; 3 APHP, Armand Trousseau Hospital, Pediatric Endocrinology, Paris, France; 4 APHP, Hôpital Armand-Trousseau, Département de Génétique, UF de Génétique Chromosomique, Paris, France; 5 Institute of Human Genetics, RWTH University Hospital Aachen, Pauwelsstr 30, D-52074 Aachen, Germany; 6 Institute of Human Genetics, Charité University Hospital, Berlin, Germany; 7 Department of Pediatrics, Icahn School of Medicine at Mount Sinai, New York, NY USA

**Keywords:** Maternal uniparental Disomy 7, Formation mechanism, Chromosome 7, Trisomic rescue

## Abstract

**Background:**

Maternal uniparental disomy (UPD) of chromosome 7 (upd(7)mat) accounts for approximately 10% of patients with Silver-Russell syndrome (SRS). For upd(7)mat and trisomy 7, a significant number of mechanisms have been proposed to explain the postzygotic formation of these chromosomal compositions, but all have been based on as small number of cases. To obtain the ratio of isodisomy and heterodisomy in UPDs (hUPD, iUPD) and to determine the underlying formation mechanisms, we analysed a large cohort of upd(7)mat patients (*n* = 73) by SNP array typing. Based on these data, we discuss the UPDs and their underlying trisomy 7 formation mechanisms.

**Results:**

A whole chromosome 7 maternal iUPD was confirmed in 28.8%, a mixture or complete maternal hUPD in 71.2% of patients.

**Conclusions:**

We could demonstrate that nondisjunction mechanism affecting chromosome 7 are similar to that of the chromosomes more frequently involved in trisomy (and/or UPD), and that mechanisms other than trisomic rescue have a lower significance than previously suspected. Furthermore, we suggest SNP array typing for future parent- and cell-stage-of origin studies in human aneuploidies as they allow the definite classification of trisomies and UPDs, and provide information on recombinational events and their suggested association with aneuploidy formation.

## Background

With a frequency of 0.5% among newborns and up to 50% among abortions, human trisomies significantly contribute to human malformations and human reproduction failure. Therefore comprehensive studies have been focused on the origin and formation mechanisms of human aneuploidies, and their etiological factors. For the common autosomal trisomies 13, 18 and 21, it has been shown that they are mainly caused by meiotic errors in oogenesis, whereas the number of trisomic cases originating from missegregation in paternal meiosis or postzygotic mitosis is low [1]. Increased maternal age as well as altered numbers and the distribution of recombination sites have been identified as risk factors for errors in the maternal meiosis (for review: [2]). Naturally, the majority of data have been obtained from the frequent human numerical aberrations, whereas studies on the other chromosomes are hampered because they are not compatible with live and therefore only randomly ascertained, e.g. in prenatal diagnosis or in abortions. As a result, it is difficult to assess whether general formation mechanisms and factors exist which contribute to chromosomal nondisjunction and trisomy, or whether these factors are specific for each chromosome as suggested by Hassold et al. [1].

**Fig. 1:**
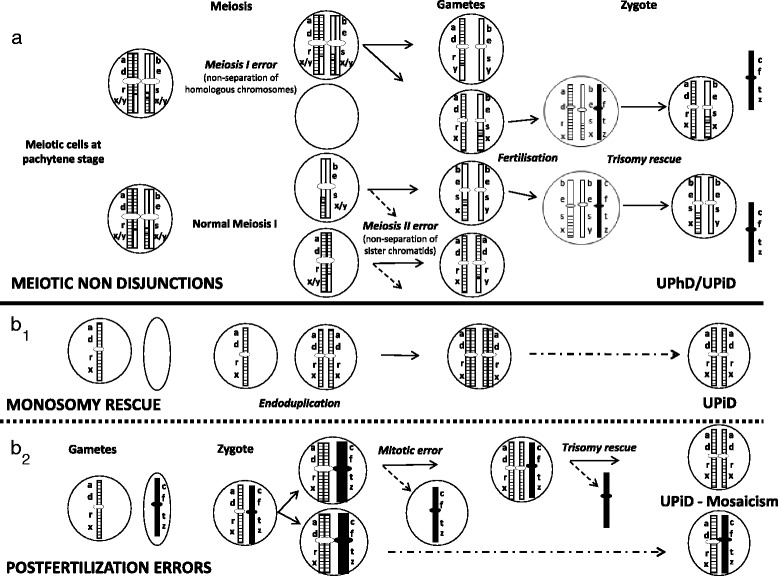
Formation mechanisms of trisomy and UPD after meiotic and mitotic nondisjunction. iUPD formation by gamete complementation is not shown as it should be very rare, but the allelic patterns correspond to those of monosomic rescue. Possible typing results of four different molecular markers are shown to illustrate the role of their physical localization (close or far from the centromere on both arms) for the discrimination between hUPD and iUPD

With the increasing number of reported cases with uniparental disomies (UPDs), this ascertainment problem could at least in part be circumvented for some of the rare trisomies in particular those which significantly contribute to the high reproductive failure in humans and/or can frequently be detected in prenatal testing (e.g. chromosomes 7, 16, 20). UPD as the exceptional inheritance of both homologues of a chromosomal pair from the same parent has been reported for nearly every human chromosome [3]. In case the affected chromosome harbors imprinted genes, an imprinting disorder will arise (e.g Prader-Willi syndrome, Silver-Russell syndrome) [4], but for many chromosomes a specific UPD phenotype does not exist as they do not harbor imprinted genes. Several mechanisms of UPD formation have been identified or suggested, with trisomic rescue as the most important one [5]: here the supernumerary chromosome of an originally trisomic zygote is lost, a mechanism which has been confirmed in-vivo (e.g. [6–9]) (Fig. 1). In contrast, other mechanisms of UPD formation are conceivable but are rare because they require a lot of events.

The parental origin and the cell-stage of formation of trisomy and UPD can be determined by polymorphic DNA markers consisting of at least two different alleles (Fig. 1). In case of a normal biparental transmission, an individual inherits one allele from each parent. In case of UPD, only alleles from one parent can be observed in the offspring. In case the contributing parent is heterozygous for two different alleles and both are transferred, the child has a uniparental heterodisomy (hUPD) for this marker. In case only one allele is inherited twice, the offspring is carrier of a uniparental isodisomy (iIUPD). By considering the physical localization of polymorphic DNA markers on a chromosome and the type of UPD (iUPD or hUPD), the stage of meiotic nondisjunction can be inferred (Fig. 1). In case pericentromeric markers show hUPD, a meiosis I error is probably the major step of UPD formation, in case of iUPD for these markers a meiosis II nondisjunction can be assumed. In contrast, a iUPD of a whole chromosomes is rather compatible with a postzygotic mitotic nondisjunction mechanism, for this constitution three different modes of formation have been postulated: (i) gamete complementation (fertilization of a nullisomic by a disomic gamete), (ii) monosomic rescue (fertilization of a nullisomic by a monosomic gamete with subsequent endoduplication), and (iii) postfertilization errors (nondisjunction in a originally disomic zygote resulting in a trisomy mosaicism and rescue in a subsequent mitosis, associated with a mosaic constitution) [5].

Until recently, nearly all studies on UPD formation have been based on microsatellite analyses (short tandem repeat markers, short sequence repeat markers), i.e. on a group of polymorphic markers consisting of alleles of different repeat numbers. The high information content of microsatellite markers and their chromosomal position allow the delineation of the parental origin of the supernumerary chromosome as well as determination of the cell-stage in which the nondisjunction occurs. However, the use of microsatellites is limited by their number and the incomplete coverage in the genome. In contrast single nucleotide polymorphisms, despite their limitation to two alleles, have the advantage of extreme frequency and wide distribution over the human genome. With the application of SNP array analysis for molecular karyotyping and the possibility to differentiate between a homozygosity and heterozygosity as well as uniparental isodisomy and heterodisomy, SNP arrays have become a valuable tool to enlighten the formation mechanisms of trisomies and UPDs [2, 10].

A relevant UPD in humans is maternal UPD of chromosome 7 (upd(7)mat), which accounts for approximately 10% of patients with Silver-Russell syndrome (SRS, [11]). It has been suggested that a considerable number of cases are the result from a postzygotically occurring trisomy 7, [1, 12], in that case trisomy 7 would be different from other autosomal trisomies. To obtain a representative overview on the ratio of isodisomy and heterodisomy in UPDs and the underlying formation mechanisms, we analysed a large cohort of upd(7)mat individuals by SNP array microsatellite typing. Based on these data, we discuss their formation mechanism and that of their underlying trisomy 7.

### Study population

Our study cohort was derived from patients who had been referred for routine testing for Silver-Russell syndrome (SRS) to either the French or the German laboratories. Some of them have already been reported [11–16]. Our final cohort consisted of the 76 patients who were confirmed to have upd(7)mat. In three patients, a segmental UPD restricted to the long arm had been reported previously [13, 14]. The others 73 patients carried a UPD of the whole chromosome 7. Upd(7)mat/upd(7q)mat was identified molecularly by microsatellite typing and methylation-specific assays targeting loci on both arms of chromosome 7.

To compare the distribution of recombination breakpoints between the German upd(7)mat carriers and controls, we used data from 28 unrelated German controls of normal growth.

The study was approved by the ethics review boards of the University Hospital of the RWTH Aachen and the Hôpitaux de Paris. Written informed consent for participation was received for all patients, either from the patients themselves or their parents.

## Methods

Upd(7)mat was identified in the routine diagnostic workup by methylation-specific tests (methylation-specific (MS)-PCR, MS single nucleotide primer extension (MS-SNuPE), MS multiplex ligation probe-dependent amplification (MS-MLPA), or ASMM RTQ-PCR (TaqMan Allele-Specific Methylated Multiplex Real-Time Quantitative PCR)), the upd(7)mat was then confirmed by microsatellite typing. Further information on the markers used and PCR conditions are available on request.

From three of the patients with a mixed hUPD/iUPD, fibroblasts were available and tested by MS-MLPA.

For genome-wide copy number analysis and to determine the size of isodisomic regions, the patients were typed by SNP array analysis.

**Fig. 2:**
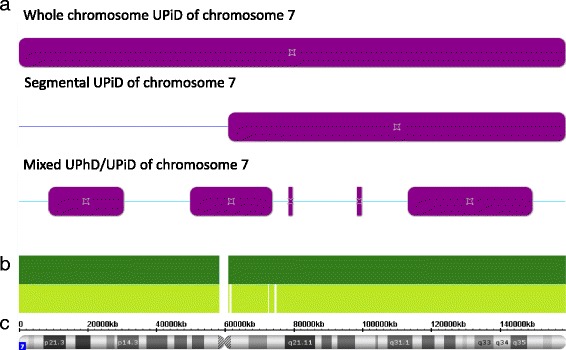
Local Affymetrix GenomeWideSNP_6.0 Array signal distribution pattern (**a**) showing total upd(7)mat, segmental iUPD(7q)mat and mixed hUPD/iUPDiUPD. Note that only a differentiation between hUPD and iUPD is possible, whereas the parental origin as well as the identification of segmental UPD is only possible by including the results of microsatellite typing. **b** Distribution of SNP (light green) and oligo probes (*dark green*). **c** Physical map of chromosome 7

In the 34 German patients, Affymetrix SNP6.0 array analysis (Affymetrix, Santa Clara, California/USA) was carried out following the manufacturer’s protocol. Data analysis was performed with the Genotyping Console and Chas software (Affymetrix). For iUPD detection, the software option “LOH” (loss of heterozygosity) was used, indicating all regions with a loss of heterozygosity (LOH) (default: >1 kb, >50 marker) (Fig. 2). Chromosomal regions were classified as iUPD in case of a LOH, the non-LOH parts of the chromosomes 7 were defined as hUPD. The number of recombinations was delineated from the number of hUPD and iUPD stretches per patient. Furthermore, mosaicism for trisomy 7 was determined by SNP array typing, using a test that had been validated for a mosaic detection level of 5%.

The 42 patients samples analysed in France were processed using Infinium assays (HumanCytoSNP-12 or Omniexpress-24BeadChips, Illumina, San Diego, California/USA) as previously described [17]. Results were analysed with the Illumina Genome Studiosoftware. For iUPD detection, CNV partition 3.1.6 software were used, indicating all regions with a loss of heterozygosity with a minimum size of 1,000,000 and a minimum 3 Probe Count.

## Results

In the group of 73 carriers of whole chromosome upd(7)mat tested by SNP array analysis, we determined a complete iUPD in 28.8% (21/73) and a mixture of complete hUPD or iUPD/hUPD in 71.2% (52/73) of patients (Table 1). Mean maternal age in the hUPD cohort (*n* = 33) was 36.24+/−5.77, in the iUPD group (*n* = 16) it was 31.33+/−5.39 years, the difference was statistically significant (*p* = 0.006).

**Table 1:** Origin and (postulated) formation mechanisms of the most frequent autosomal trisomies and UPDs. (°only UPD cases with a definite classification as hUPD or iUPD from [18] are listed)

Chromosome	N=	Trisomy					N=	UPD°				Reference
		Maternal		Paternal		PZM		Maternal		Paternal		
		MI	MII	MI	MII			UPhD	UPiD	UPhD	UPiD	
2	18	53.4%	13.3%	27.8%		5.6%	11	45.4%	36.4%	9.1%	9.1%	Reviewed by [1, 3]
6							18	-	11.1%		88.8%	Reviewed by [3]
7	14	27.2%	25.7%			57.1%	55	61.8%	38.2%		4.3%	Reviewed by [1, 18]
							73	71.2%	28.8%			Own data: [11, 12]
8	12	50.0%	50.0%			50.0%	4	50.0%	25.0%		25.0%	Reviewed by [1, 3]
13	74	56.6%	33.9%	2.7%	5.4%	1.4%	10	30.0%	20.0%	10.0%	40.0%	Reviewed by [1, 3]
14	26	36.5%	36.5%		19.2%	7.7%	48	45.8%	28.8%	10.4%	17.7%	Reviewed by [1, 3]
15	34	76.3%	9.0%		14.7%		62	80.6%	6.5%	1.6%	11.3%	Reviewed by [1, 8]
16	104	100.0%					35	91.4%	5.7%		2.8%	Reviewed by [1, 3]
18	150	33.3%	58.7			8.0%	2					Reviewed by [1, 3]
20	3	2		1		-	5	60.0%	20.0%		20.0%	Reviewed by [3]
21	782	69.6%	23.6	1.7%	2.3%	2.7%	12	33.3%	33.3%		33.3%	Reviewed by [1, 3]
22	130	86.4%	10.0	1.8%		1.8%	17	52.9%	11.8%		35.3%	Reviewed by [1, 3]

SNP array analysis in the three segmental upd(7q)mat carriers revealed a iUPD for the whole uniparental regions and confirmed the sizes of the UPD segments obtained from previous microsatellite studies [13, 14].

The frequency of recombination events was determined in 20 German hUPD carriers by the Affymetrix SNP 6.0 array analysis. On average, 9.2 recombinations/chromosome 7 could be observed per individual, this frequency did not differ significantly from that in the control population (9.8/individual). Furthermore, the recombination events showed a similar distribution over the whole chromosome 7 in both cohorts.

**Fig. 3:**
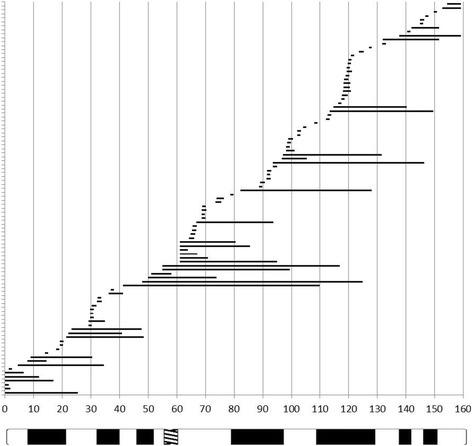
Analysis of the data from the array typing in hUPD carriers: Distribution of uniparental isodisomy stretches in the 20 hUPD cases analysed by the Affymetrix SNP6.0 arrays

In the hUPD cohort, there was no common isodisomic region but the isodisomic regions were randomly distributed (Fig. 3).

Array typing in lymphocytes as well as MS-MLPA in fibroblasts from three out of these cases did not provide evidence for a trisomy 7/upd(7)mat mosaicism.

As expected, molecular karyotyping revealed several apathogenic copy number variations, but there was no evidence for any pathogenic genomic imbalances in this cohort.

## Discussion

Previous reports suggested that in upd(7)mat and trisomy 7 formation postzygotic mitotic nondisjunction plays a significant role, accounting for 40 to 57% of cases, respectively (Table 1), [1, 3, 12, 18, 19] Based on the heterogeneous findings in trisomies and UPDs of other chromosomes it has been suggested that three chromosome-specific nondisjunction mechanisms should exist: a) those accounting for all chromosomes, b) those affecting a subset of chromosomes, and c) those responsible for aneuploidies of single chromosomes [1]. However, some of these assumptions were based on single case reports or small cohorts, and a standardized set of markers covering a whole chromosome has not been applied. With the present study we can show for chromosome 7 that the majority of whole chromosome upd(7)mat cases are hUPD or mixed hUPD/iUPD and thus originates from maternal meiosis nondisjunction. With a percentage of ~71%, maternal meiotic errors are the dominant cause of upd(7)mat formation, corresponding to that of the common autosomal trisomies (Table 1). In fact, this number is probably higher, as some iUPD might originate from a meiotic II error without precedent recombination. Additionally, the major role of maternal meiosis in upd(7)mat formation is corroborated by the increased maternal age in the hUPD group in comparison to the iUPD cohort. Altered numbers and locations of recombination events as further factor contributing to nondisjunction in trisomy 21 could not be confirmed for upd(7)mat.

Interestingly, we did not get any evidence for trisomy 7 mosaicism in three hUPD carriers, although the upd(7)mat formation by trisomic rescue has been proven directly or indirectly in single studies [6, 7, 12, 20, 21]. In fact, low-level mosaicism can hardly be detected by SNP array analysis, but we think that trisomy 7 cell lines should be extremely rare in the body as this constitution is not compatible with life. The situation seems to be different in the placenta as the presence of extraembryonic trisomy 7 cells does obviously not affect fetal growth [22]. As a result, it is obvious that the SRS phenotype is associated with the upd(7)mat and not with an undetectable trisomy 7.

Trisomic rescue after meiotic nondisjunction is probably the major mode of upd(7)mat formation in case of hUPD. However, in case of iUPD the formation mechanism is difficult to determine. At first glance, the reports on extraembryonic trisomy 7 mosaicism in upd(7)mat indicate that a postzygotic nondisjunction followed by a trisomic rescue might have occurred. In that case mosaicism for a normal biparental disomic, a trisomic and iUPD cell line can be expected. As there is no functional reason for the elimination of the biparental disomic cells, iUPD should therefore be associated with a mosaicism for at least iUPD and normal cells. However, there is no evidence for a iUPD/biparental cell lines mosaicism, we therefore assume that whole iUPD of chromosome 7 rather occurs by monosomy rescue than by a postfertilization error.

In the three carriers of a segmental upd(7q)mat we could confirm that the uniparental segments were totally isodisomic. This finding is in agreement with similar studies on segmental UPDs of chromosomes 6 and 11 representing the major fraction of reported segmental UPDs in humans [23] (for review: [24, 25]). Interestingly, mosaicism for segmental UPD can be observed only in BWS, caused by postzygotic mitotic errors. In case of segmental upd(6q)mat and upd(7q)mat a mosaic distribution has not yet been reported, it therefore remains unclear whether they are also caused mitotically or by meiotic errors.

In our cohort of 73 upd(7)mat carriers we did not detect any further uniparental (iso)disomies or pathogenic copy number variations, as reported recently for a maternal upd(6)mat and a upd(11p)pat carrier [10, 14]. Furthermore, there was no evidence for a common isodisomic segment on chromosome 7 in all patients (Fig. 3). Thus, an autosomal recessive factor on this chromosome causing SRS can be excluded, and we thereby support the microsatellite-based data from Preece et al. [26].

Our results confirm the power of SNP array typing to characterize hUPDs and to determine UPD formation mechanisms. As Keren et al. [23] recently demonstrated, SNP array analysis is also a valuable tool to determine the mosaic distribution of upd(11)pat and partial trisomy 11 cell lines in BWS. However, in case of upd(7)mat mosaicism both of UPD and trisomic cell lines is extremely rare. Therefore for routine diagnosis, it is sufficient to perform methylation-specific tests and microsatellite typing.

## Conclusions

Our data illustrate the limitations of microsatellite-based formation studies, thus results based on this type of markers should be considered with caution as they depend on the distribution of the used markers over the chromosome. Rather we suggest SNP array typing for future parent- and cell-stage-of origin studies in human aneuploidies, because they allow the definite classification of trisomies and UPDs. Additionally, they provide information on recombinational events and their suggested association with aneuploidy formation [2].

Using this approach, we demonstrated that nondisjunction mechanisms affecting chromosome 7 are similar to those affecting chromosomes more frequently involved in trisomy (and/or UPD), and that mechanisms other than trisomic rescue have a lower significance than previously suspected.
